# Fixed complex electrograms during sinus rhythm and local pacing: potential ablation targets for persistent atrial fibrillation

**DOI:** 10.1038/s41598-022-14824-4

**Published:** 2022-06-23

**Authors:** Buyun Xu, Chao Xu, Yong Sun, Jiahao Peng, Fang Peng, Weiliang Tang, Yan Zhou, Shengkai Wang, Jie Pan, Yangbo Xing

**Affiliations:** 1 Department of Cardiology, Shaoxing People’s Hospital (Shaoxing Hospital, Zhejiang University School of Medicinel), 568 # Zhongxing North Road, Shaoxing, 312000 Zhejiang People’s Republic of China; 2 Loma Linda University School of Public Health, 24951 Circle Dr, Loma Linda, CA 92354 USA

**Keywords:** Cardiology, Interventional cardiology

## Abstract

In atrial fibrillation (AF) patients, complex electrograms during sinus rhythm (C-EGMs) could be pathological or not. We aimed to demonstrate whether local pacing was helpful to discern pathological C-EGMs. 126 persistent AF patients and 27 patients with left-side accessory pathway (LAP) underwent left atrial mapping during sinus rhythm. If C-EGMs were detected, local pacing was performed. If the electrograms turned normal, we defined them as non-fixed C-EGMs, otherwise as fixed C-EGMs. No difference was detected in the incidence and proportion of non-fixed C-EGMs between AF patients and LAP patients (101/126 vs. 19/27, *P* = 0.26; 9.1 ± 6.0% vs. 7.7 ± 5.7%, *P* = 0.28). However, the incidence and proportion of fixed C-EGMs were higher in persistent AF patients (87/126 vs. 1/27, *P* < 0.01; 4.3 ± 3.4% vs. 0.1 ± 0.5%, *P* < 0.01). Compared with non-fixed C-EGMs, fixed C-EGMs had lower amplitudes, longer electrogram durations and longer Stimuli-P wave internals. All AF patients received circumferential pulmonary vein isolation. Among AF patients with fixed C-EGMs, 45 patients received fixed C-EGMs ablation and 42 patients underwent linear ablation. Compared with linear ablation, fixed C-EGMs ablation reduced recurrence (HR: 0.43; 95% CI 0.21‐0.81; *P* = 0.011). Among patients without fixed C-EGMs ablation, the proportion of fixed C-EGMs was an independent predictor of ablation outcomes (HR for per percent: 1.13, 95% CI 1.01–1.28, *P* = 0.038). C-EGMs could be classified into fixed and non-fixed C-EGMs through local pacing. Fixed rather than non-fixed C-EGMs might indicate abnormal atrial substrates and fixed C-EGMs ablation improve outcomes of persistent AF ablation.

## Introduction

Catheter ablation based on circumferential pulmonary vein isolation (CPVI) has been recommended as the first-line treatment for symptomatic paroxysmal atrial fibrillation (AF), with a 1-year success rate of ~ 70%. However, for persistent AF, the 1-year success rate of simple CPVI is only ~ 40–50%^1^. Therefore, additional ablation strategies beyond CPVI are required for persistent AF. In the past two decades, several adjunctive strategies have been explored to improve the outcomes of catheter ablation for persistent AF, including complex fractionated atrial electrograms ablation, linear ablation, low voltage area ablation and rotors ablation^2^. Unfortunately, no ablation strategy achieved satisfactory results in large randomised controlled trials. Therefore, for persistent AF, it is necessary to develop a new ablation strategy that does beyond CPVI to reduce recurrence after catheter ablation.

Extracellular electrograms (EGMs) depict the electrical activity of the underlying myocardium^3^. Recently, several studies demonstrated that complex electrograms recorded during sinus rhythm (C-EGMs) were associated with some pathological mechanisms related to AF, including myocardial fibrosis, parasympathetic innervation, heterogenetic gap junction and no-pulmonary foci^4–8^. Therefore, in theory, C-EGMs were potential ablation targets during persistent AF ablation. However, several studies demonstrated that approximately 40–80% C-EGMs were functional^9–12^.

Wave fronts collision and far-field potential were the major causes of non-pathological C-EGMs^9–12^. We hypothesized that pacing beside the points with C-EGMs could avoid wave-front collision at these points. In addition, local pacing could help to identify far-field potential. If these hold true, most non-pathological C-EGMs would become unfractionated in the setting of local pacing and it would be helpful to recognize pathological C-EGMs which might be potential ablation targets.

## Methods

### Study patients

From June 2018 to June 2020, we enrolled consecutive patients undergoing catheter ablation for persistent AF at Shaoxing People's Hospital were enrolled. Persistent AF is defined according to the European society of Cardiology Guidelines for AF^1^. To evaluate the characteristics of non-pathological C-EGMs, we also enrolled patients undergoing left-sided accessory pathway ablation without structural heart disease or a history of AF. Patients with previous ablation of arrhythmia, valvular disease, hypertrophic cardiomyopathy, severe pulmonary disease, hyperthyroidism and severe liver or renal dysfunction were excluded from the study. All patients provided written informed consent, and the study was conducted in accordance with the declaration of Helsinki. The Ethics Committee of Shaoxing People's Hospital approved the study design.

### Mapping procedure

All antiarrhythmic drugs were discontinued for at least five half-lives and amiodarone was discontinued for at least 2 weeks before study initiation. All AF patients received a transesophageal echocardiography to exclude thrombi, within 48 h before undergoing ablation. Electrophysiological study was performed with the patient sedated using midazolam and fentanyl. A 6F decapolar catheter was inserted into the coronary sinus via the left femoral vein. A circular mapping catheter (10-pole, 1.35 mm ring electrode with 3.5-mm interelectrode spacing, A-Focus II catheter, St. Jude Medical) and an irrigated ablation catheter were introduced into the left atrium (LA) through two transseptal punctures. Three dimensional geometries of the LA and PVs were created by the circular mapping catheter and Ensite-NavX (St. Jude Medical, St. Paul, MN, USA).

**Figure 1:**
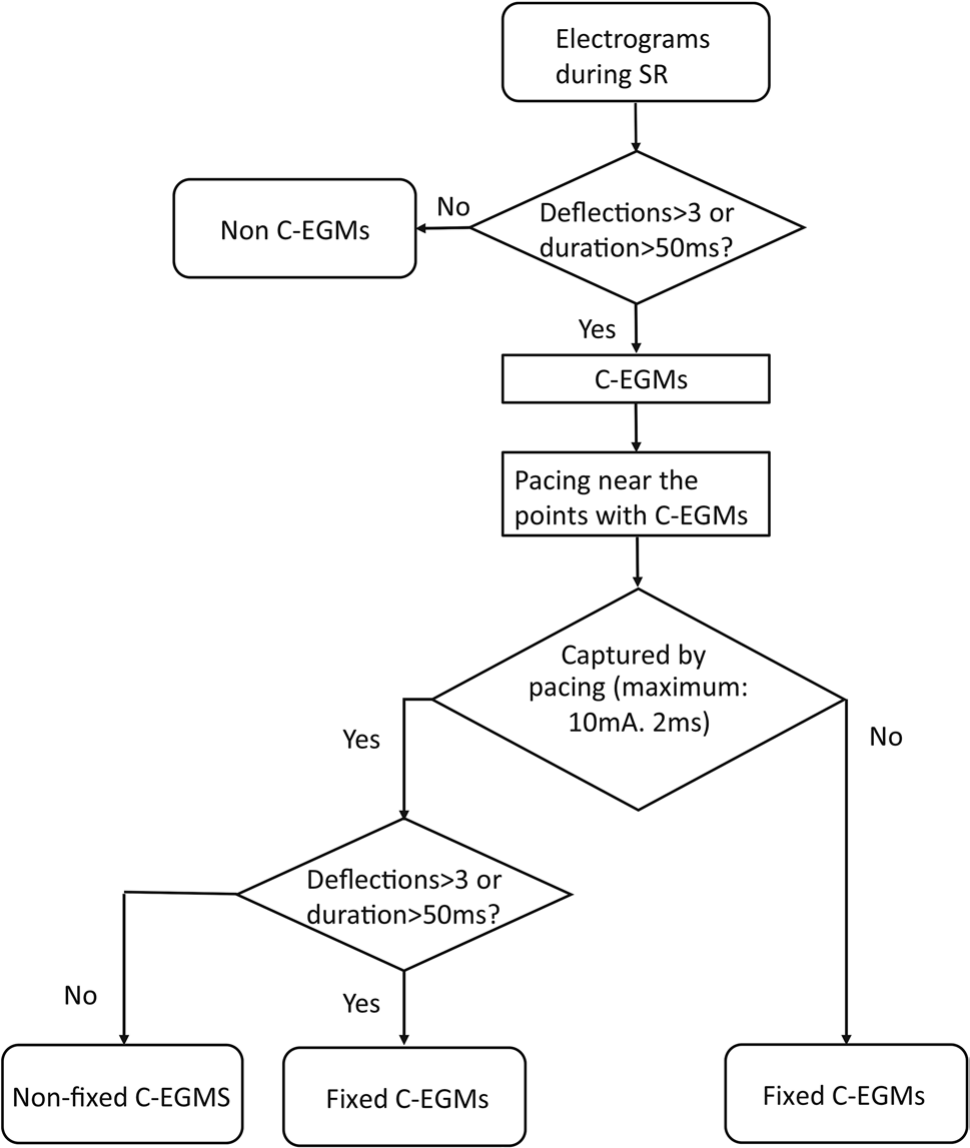
Identification of C-EGMs, fixed C-EGMs and non-fixed C-EGMs. SR, sinus rhythm; C-EGMs, complex electrograms in sinus rhythm.

**Figure 2:**
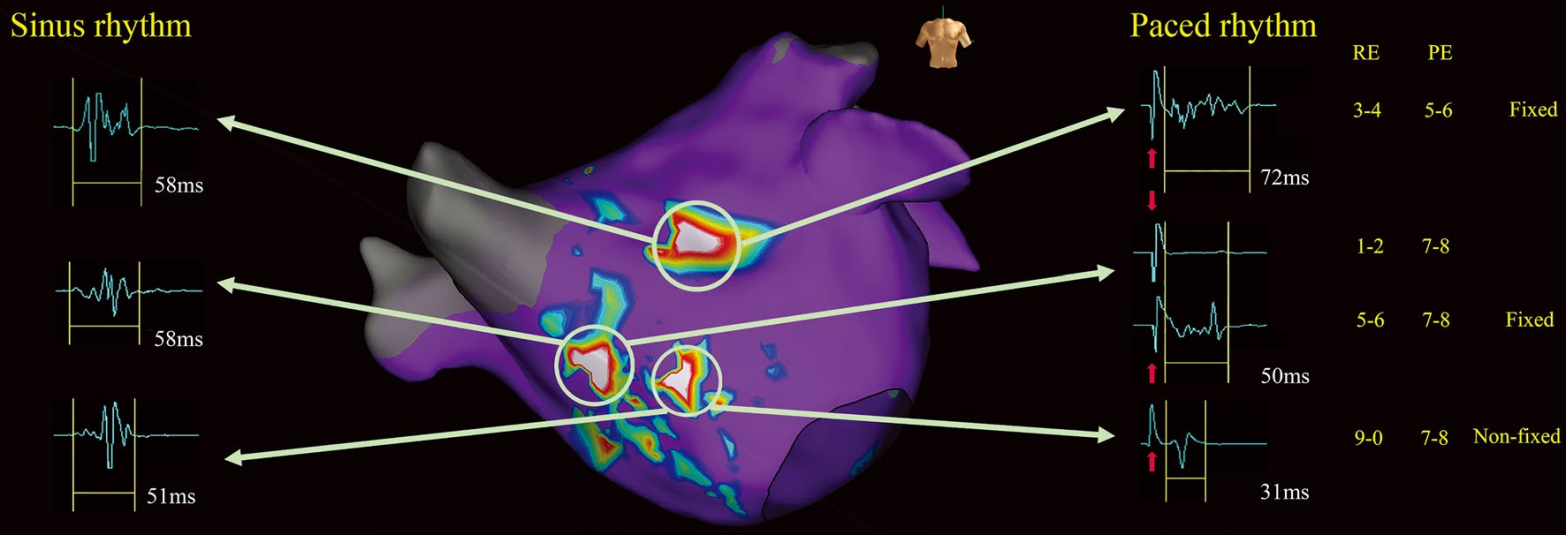
Examples of fixed C-EGMs and non-fixed C-EGMs. Left: Electrograms recorded during sinus rhythm. Middle: A left atrium sinus rhythm fractionation map in anteroposterior view. Right: Electrograms recorded during paced rhythm. Red arrows indicated pacing artifacts. Note the middle electrograms. Pacing artifact merged with the electrogram of interest (electrodes 5–6) and it was difficult to identify the onset of the electrogram. The pacing artifact recorded in electrodes 1–2 was clear and was used as a reference to measure the electrogram of interest. (Graphics software: Ensite Velocity and Microsoft Office PowerPoint v16.0). C-EGMs, complex electrograms in sinus rhythm; PE, pacing electrode; RE, recording electrode.

To avoid the effect of ablation, C-EGMs mapping was performed before ablation^13^. If the rhythm was AF before mapping, cardioversion was conducted to restore sinus rhythm. After a waiting-period of at least 5 min, a detail bipolar electroanatomic map of the LA (filter setting, 30-500 Hz) was created by two operators with over 5 years’ experience in cardiac electrophysiology study. The peak-peak sensitivity threshold was set to 0.05 mV. At least 300 points (excluding points within the oval foramen, pulmonary veins and pulmonary vein antrum) were required to create each map and care was taken to ensure an even distribution of the points. At least five continuous beats with stable EGMs were required for each point. Points beyond 5 mm from the geometry shell were excluded and premature atrial beats were eliminated. Complex electrograms were tagged manually according to the definition as follows: (1) the number of deflections > 3, counted as the number of positive or negative peaks; or (2) EGMs duration > 50 ms^14^. If C-EGMs were recorded, bipolar pacing from a neighbouring electrode of the same circular mapping catheter was performed (for example, if C-EGMs were recorded from electrodes 5–6, electrodes 7–8 or 3–4 could be used as pacing electrodes). To avoid points with C-EGMs from being captured directly by pacing stimuli, we used the pacing threshold as the pacing stimulus strength. Sites at which the pacing threshold exceeded 10 mA with 2 ms width were regarded as electrically unexcitable tissue. The pacing interval was set as the RR interval during sinus rhythm minus 30 ms. During paced rhythm, at the points with C-EGMs, more than five continuous captured beats with stable EGMs were recorded. If the EGMs still fulfilled the criteria of complex electrograms, we defined the C-EGMs as fixed C-EGMs. If the morphology of the EGMs normalised, the C-EGMs were classified as non-fixed C-EGMs. If the points with C-EGMs could not be captured by 10-mA pacing stimuli, we also defined the C-EGMs as fixed C-EGMs. The definition and classification of C-EGMs are summarized in Fig. 1. A representative example of fixed C-EGMs and non-fixed C-EGMs is shown in Fig. 2.

In some cases, EGMs were merged with pacing artifacts. It was difficult to identify the onset of EGMs which made the electrogram durations unmeasurable. In this setting, pacing artifacts recorded from electrodes distant from pacing sites were used as references that could be clearly identified. We measured the duration of the EGMs starting from the end of the referenced pacing artifacts to the end of the EGMs (Fig. 1).

### Quantitative analysis

Points within the oval foramen, pulmonary veins and pulmonary vein antrum (0.5–1 cm extending from the pulmonary vein orifice) were excluded. The proportion, distribution and characteristics of C-EGMs were analyzed by two investigators who were. All disagreements between the two investigators were resolved by discussion.

**Figure 3:**
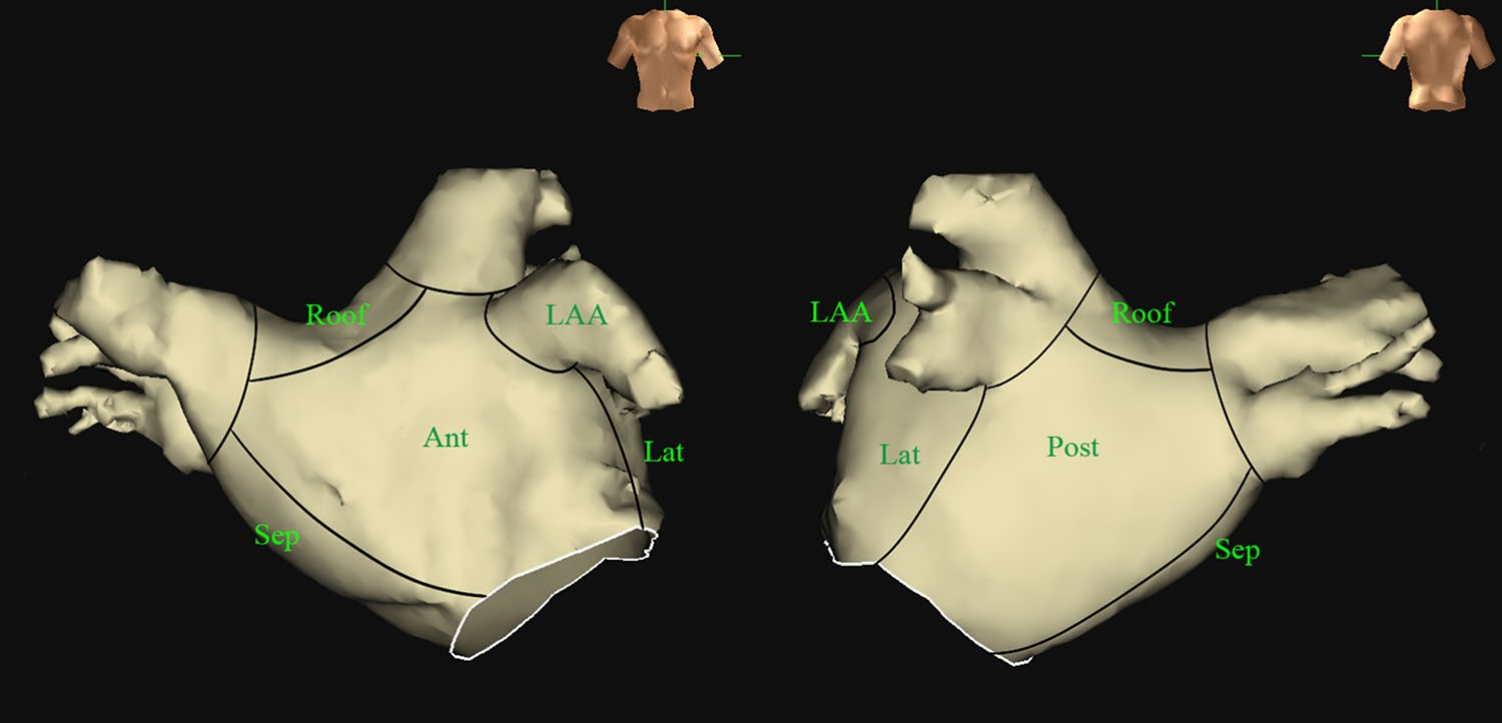
Segment of left atrium. (Graphics software: Ensite elocity). Ant, anterior wall; Lat, lateral wall; LAA, left atrial appendage; Post, posterior wall; Sep, septum.

The proportions of C-EGMs were calculated as the number of points with C-EGMs divided by the total number of points obtained. The LA surface was divided into six segments for distribution analysis: roof, anterior wall, posterior wall, septum, lateral wall, and LA appendage (Fig. 3). To comprehensively evaluate the distribution of C-EGMs, the incidence of C-EGMs in each segment was calculated. Moreover, we also evaluated the relationship between C-EGMs and low voltage area (LVA) (< 0.4 mV) and transitional voltage zones (0.4–1.3 mV)^15^.

The characteristics of the C-EGMs, including amplitudes and durations were measured. The amplitude of the EGMs was the voltage difference measured from the most negative peak to the most positive. The duration of C-EGMs was defined as the time from onset of EGMs to the time of final return to baseline. In addition to the morphological characteristics, we also measured the interval between stimuli, which were delivered directly at the points with fixed C-EGMs, and the onset of the P-wave (S-P interval).

### Ablation procedure

All patients received CPVI. Either fixed C-EGMs ablation or linear ablation (anterior line, roof line, and mitral isthmus line) was performed at the operators’ discretion. The ablation parameters were as follows: target temperature: 45°, maximum power: 35–40 W, infusion rate: 17 mL/min. Procedural endpoints were bidirectional conduction block of all PVs and linear lesions (if linear ablation were performed), and a decrease of fixed C-EGMs amplitude by 80% (if fixed C-EGMs ablation were performed). After ablation, amiodarone was prescribed for three months.

### Follow-up

After ablation, patients were followed-up regularly at 1, 3, 6, and 12 months after ablation and every 6 months after the first year. A blanking period of 3 months was considered for the study. Follow-up visit included intensive questioning for arrhythmia-related symptoms, ECG and 24 h Holter recording. All documented episodes of atrial tachycardia (AT), atrial flutter (AFL) and AF lasting ≥ 30 s were considered as a recurrence.

### Statistical analysis

Continuous data were expressed as mean ± SD or median (minimum, maximum), according to normality test. Normal distribute data were compared using the Student’s *t*-test or one-way analysis of variance (ANOVA). Tukey’s or Dennett’s tests were used for multiple comparisons. Skewed data were compared using the Mann–Whitney test. Categorical data were expressed as counts and were compared using Pearson's χ^2^ test or Fisher exact test. Survival was evaluated using a Kaplan–Meier analysis. Multivariate Cox proportional-hazards regression with forward likelihood ratio was performed to identify independent risk factors for the occurrence of endpoint events. All potential confounders based on known clinical relevance and parameters that had a *P*-value < 0.20 in univariate analyses were entered into the model. All data were analyzed using SPSS 24.0. Statistical significance was set at *P* < 0.05.

## Results

The data supporting the findings of this study are available from corresponding author upon reasonable request.

### Characteristics of participants

During the study period, a total of 138 patients with persistent AF were recruited. Twelve patients were excluded because sinus rhythm could not be maintained for sufficient time to complete the mapping procedure. Therefore, 126 patients with persistent AF were included in the analysis. The mean age of enrolled AF patients was 65 ± 8 years and 81/126 (64.3%) patients were male. The mean duration of AF was 27.1 ± 24.1 months. Besides AF patients, 27 patients with left-side accessory pathway (LAP) were enrolled. Compared to AF patients, LAP patients were younger (44 ± 15 years), and had smaller left atrial anteroposterior diameters (40.4 ± 5.3 vs. 32.3 ± 4.1 mm, *P* < 0.01), a lower incidence of hypertension (79/126 vs. 3/27, *P* < 0.01) and lower CHA_2_DS_2_-VASc scores (2.0 ± 1.3 vs. 0.6 ± 0.6, *P* < 0.01). Characteristics of participants are summarized in Table 1.

**Table 1:** Characteristics of participants.

	Persistent AF (n = 126)	LAP Group (n = 27 )	*P* value
Age (years)	65 ± 8	44 ± 15	< 0.01
Male (n)	81 (64.3%)	17 (63.0%)	0.90
BMI (kg/m^2^)	24.4 ± 3.1	22.6 ± 2.6	< 0.01
Duration of AF (months)	27.1 ± 24.1	–	–
Hypertension (n)	79 (62.7%)	3 (11.1%)	< 0.01
Diabetes (n)	24 (19.0%)	1 (3.7%)	0.08
CHD (n)	10 (7.9%)	0 (0.0%)	0.21
Heart failure (n)	8 (6.3%)	0 (0.0%)	0.35
CHA_2_DS_2_-VASc score	2.0 ± 1.3	0.6 ± 0.6	< 0.01
eGFR (ml/min)	89 ± 15	102.0 ± 12	< 0.01
LAD (mm)	40.4 ± 5.3	32.3 ± 4.1	< 0.01
LVEF (%)	64 ± 7	68 ± 5	0.03

AF, atrial fibrillation; BMI, body mass index; CHD, coronary heart disease; eGFR, estimated glomerular filtration rate; LAD, left atrial dimension; LAP, left-side accessory pathway; LVEF, left ventricular ejection fraction.

### Incidence and proportion of C-EGMs

**Figure 4:**
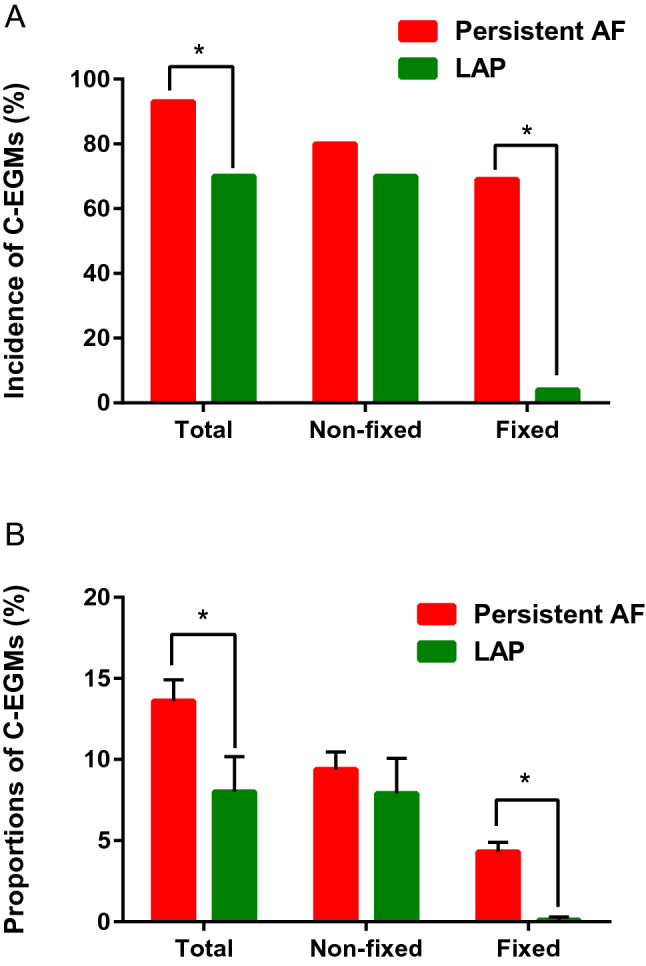
(**A**) Incidence and (**B**) proportion of complex electrograms. *P < 0.01. AF, atrial fibrillation; C-EGMs, complex electrograms in sinus rhythm; LAP, left-side accessory pathway.

An average of 473 ± 90 and 393 ± 69 points were evaluated per patient in the persistent AF and LAP group, respectively. The incidence and proportion of C-EGMs was significantly higher in patients with persistent AF (117/126 vs. 19/27, *P* < 0.01; 13.4 ± 6.7% vs. 8.0 ± 5.5%, *P* < 0.01). During paced rhythm, C-EGMs could be classified as fixed and non-fixed C-EGMs. There was no difference in the incidence and proportion of non-fixed C-EGMs between the two groups (101/126 vs. 19/27, *P* = 0.26; 9.1 ± 6.0% vs. 7.7 ± 5.7%, *P* = 0.28). However, regarding fixed C-EGMs, a significant difference existed in the incidence and proportion (87/126 vs. 1/27, *P* < 0.01; 4.3 ± 3.4% vs. 0.1 ± 0.5%, *P* < 0.01). In the LAP group, fixed C-EGMs were recorded in only one patient who had left atrium remodeling (left atrial anteroposterior dimension: 42 mm) and the proportion of fixed C-EGMs was only 2.7%. The incidence and proportion of C-EGMs are illustrated in Fig. 4.

### Distribution and characteristics of C-EGMs

**Figure 5:**
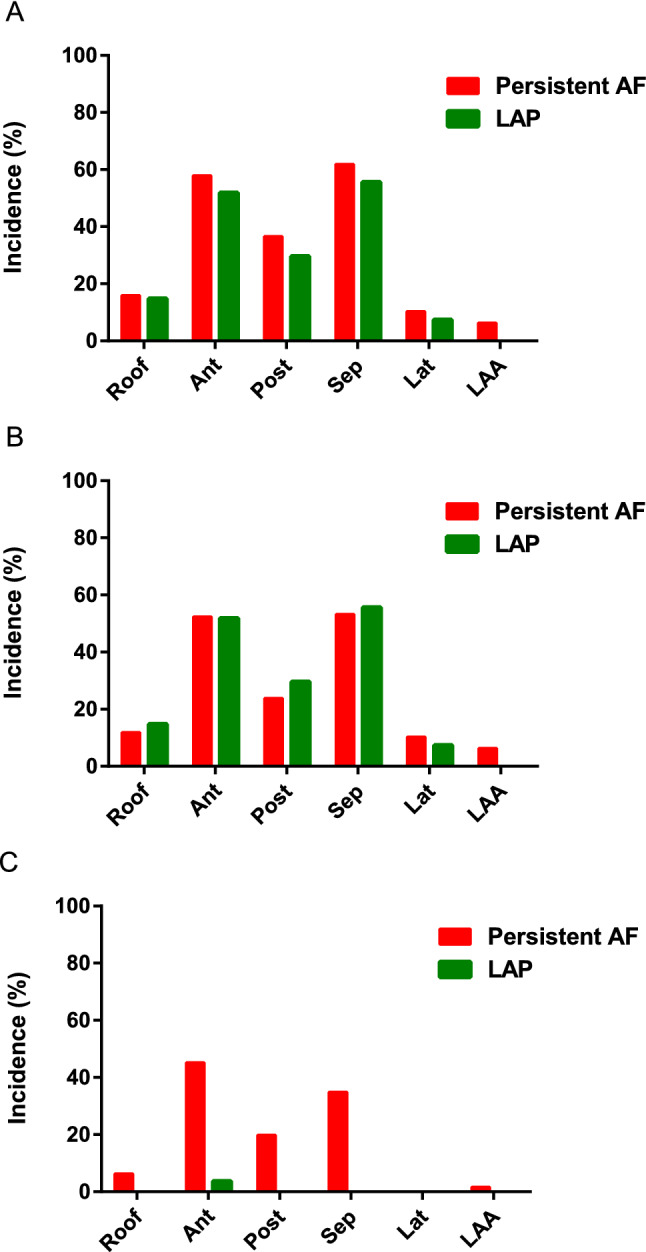
(**A**) Distribution of C-EGMs, (**B**) non-fixed C-EGMs and (**C**) fixed C-EGMs. Ant, anterior wall; Lat, lateral wall; LAA, left atrial appendage; Post, posterior wall; Sep, septum; AF, atrial fibrillation; C-EGMs, complex electrograms in sinus rhythm; LAP, left-side accessory pathway.

**Figure 6:**
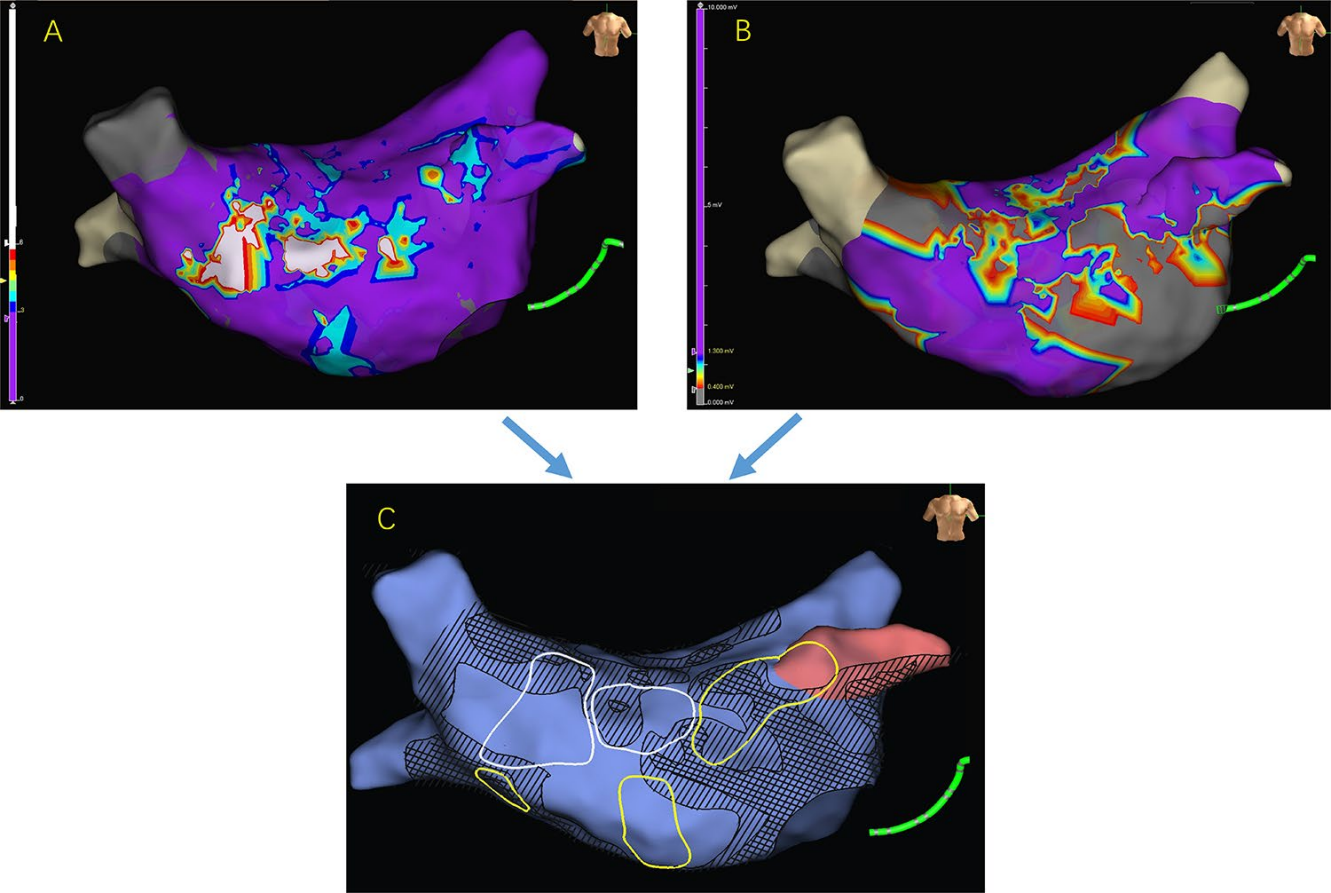
The relationship between C-EGMs and low voltage zones and transitional voltage zones. (**A**) a C-EGMs map of left atrium; (**B**) a voltage map of left atrium in the same patient; (**C**) the overlap between the C-EGMs region (the white circle indicate the fixed C-EGMs region and the yellow circle indicate the no-fixed C-EGMs region) and low voltage zones (grid areas) and transitional voltage zones (slashed areas). (Graphics software: Ensite Velocity and Microsoft Office PowerPoint v16.0).

Non-fixed C-EGMs were most prevalent in the left atrial septum (54%) and anterior wall (52%) without significant difference between the two groups. In patients with persistent AF, the incidence of fixed C-EGMs was highest on the LA anterior wall (45%), LA septum (35%), and LA posterior wall (20%). The distribution of C-EGMs is summarized in Fig. 5. In addition, in AF patients, the proportion of C-EGMs overlapping with LVA was similar between fixed and non-fixed C-EGMs (7.5 ± 7.9% vs. 6.3 ± 5.4%, *P* = 0.45). But fixed C-EGMs were more likely detected in transitional voltage zones than non-fixed C-EGMs (53.7 ± 21.1% vs. 31.7 ± 18.3%, *P* < 0.01). An example was shown in Fig. 6.

In AF patients, compared with non-fixed C-EGMs, the mean voltage of fixed C-EGMs was lower (0.81 ± 0.43 mV vs. 1.17 ± 0.73 mV, *P* < 0.01). The mean duration of fixed C-EGMs was longer than non-fixed C-EGMs (69.3 ± 18.2 ms vs. 53.8 ± 11.6 ms, *P* < 0.01). And the S-P wave interval was also significantly longer in the region expressing fixed C-EGMs than non-fixed C-EGMs (54.0 ± 23.1 ms vs. 22.3 ± 18.7 ms, *P* < 0.01).

### Outcomes of ablation

**Figure 7:**
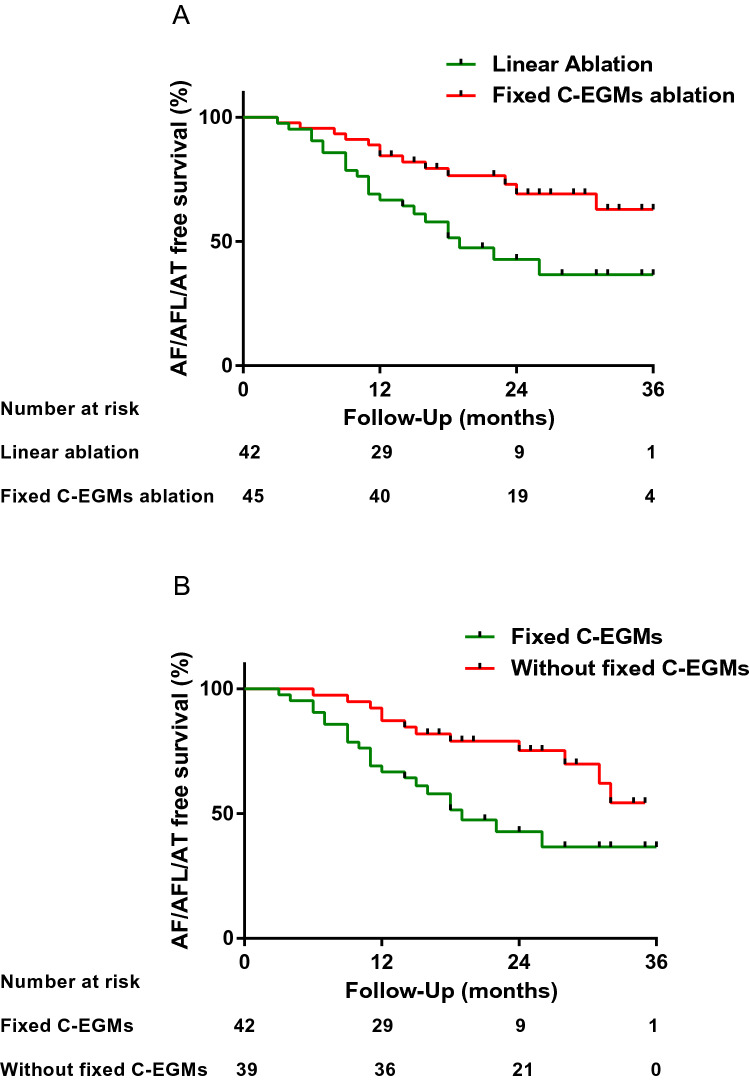
The Kaplan‐Meier curve comparing AT/AFL/AF free survival after AF ablation. (**A**) Among AF patients with fixed C-EGMs, fixed C-EGMs ablation significantly decreased incidence of recurrence compared with linear ablation (HR: 0.43; 95% CI 0.21‐0.81; *P* = 0.011 by log‐rank test). (**B**) In AF patients receiving linear ablation, patients without fixed C-EGMs were associated with a lower relative risk for recurrence (HR: 0.41, 95% CI 0.20–0.79, *P* = 0.009 by log‐rank test). AF, atrial fibrillation; AFL, atrial flutter; AT, atrial tachyarrhythmia; C-EGMs, complex electrograms in sinus rhythm; CI, confidence interval; HR, hazard ratio.

Among persistent AF patients with fixed C-EGMs, 45 patients received fixed C-EGMs ablation (group A). The remaining 42 patients underwent linear ablation (group B). The patients’ characteristics including duration of AF, left atrium size, CH2ADS2-Vasc scores and proportions of C-EGMs were balanced between the two groups. The ablation duration was shorter and RF applications were fewer in group A than in group B (Table 2). During a median follow-up of 25 months (12–36) months, AT/AFL/AF recurrence was significantly lower in the group A (13/45 vs. 22/42, *P* = 0.03). In the Kaplan‐Meier analysis, compared with linear ablation, fixed C-EGMs ablation was significantly associated with a lower recurrence risk (hazard ratio [HR]: 0.43; 95% confidence interval [CI]: 0.21‐0.81; *P* = 0.011 by log‐rank test) (Fig. 7A). After exclusion of AT/AFL recurrence (1 in the group A and 5 in the group B), fixed C-EGMs ablation still contributed to a lower recurrence rate (HR: 0.47; 95% confidence interval [CI]: 0.21‐0.94; *P* = 0.036 by log‐rank test).

**Table 2:** Characteristics of persistent AF patients.

	Group A^a^ (n = 45)	Group B^b^ (n = 42)	Group C^c^ (n = 39)	*P* value	Groups with pairwise significant difference
Age (years)	66 ± 9	64 ± 7	63 ± 7	0.18	–
Male (n)	28 (62.2%)	29 (69.0%)	24 (61.5%)	0.73	–
BMI (kg/m^2^)	25.1 ± 2.8	24.3 ± 3.2	23.8 ± 3.3	0.16	–
Duration of AF (months)	33.2 ± 23.9	28.3 ± 26.5	18.8 ± 19.4	0.02	A versus C
Hypertension (n)	30 (66.7%)	30 (71.4%)	19 (48.7%)	0.09	–
Diabetes (n)	9 (20.0%)	11 (26.2%)	4 (10.3%)	0.19	–
CHD (n)	4 (8.9%)	6 (14.3%)	0 (0%)	0.24	–
Heart failure (n)	3 (6.7%)	4 (9.5%)	1 (2.6%)	0.40	–
CHA_2_DS_2_-VASc score	2.3 ± 1.3	2.1 ± 1.3	1.6 ± 1.1	0.04	A versus C
eGFR (ml/min)	87 ± 18	90 ± 12	89 ± 13	0.35	–
LAD (mm)	42.1 ± 6.5	41.2 ± 3.9	37.6 ± 3.9	< 0.01	A versus C, B versus C
LVEF (%)	64 ± 7	63 ± 8	66 ± 6	0.17	–
Follow-up (months)	24 (12, 36)	24 (13, 36)	26 (14, 36)	0.20	–
**Linear ablation**					
Anterior line	–	34 (81.0%)	31 (79.5%)	0.87	–
Roof line	–	42 (100%)	39 (100%)	–	–
Mitral isthmus line	–	20 (47.6%)	17 (43.6%)	0.72	–
Acute success of linear ablation	–	41 (97.6%)	37 (94.9%)	0.61	–
**Procedure parameters**					
Non-fixed C-EGMs (%)	9.1 ± 5.4	8.7 ± 5.8	9.4 ± 6.9	0.87	–
Fixed C-EGMs (%)	6.3 ± 2.2	6.1 ± 2.4	–	0.70	–
Mapping duration (min)	38.0 ± 6.5	39.0 ± 8.4	33.6 ± 6.7	< 0.01	A versus C, B versus C
Ablation duration (min)	7.1 ± 2.7	23.1 ± 6.3	20.6 ± 4.9	< 0.01	A versus B, A versus C
Application of RF (n)^d^	16 ± 7	40 ± 11	36 ± 10	< 0.01	A versus B, A versus C

^a^Persistent AF patients with fixed-C-EGMs and received fixed-C-EGMs ablation were classified as Group A.

^b^Persistent AF patients with fixed-C-EGMs and received linear ablation were classified as Group B.

^c^Persistent AF patients without fixed-C-EGMs and received linear ablation were classified as Group C.

^d^Excluding circumferential pulmonary vein isolation.

AF, atrial fibrillation; BMI, body mass index; C-EGMs, complex electrograms during sinus rhythm; CHD, coronary heart disease; eGFR, estimated glomerular filtration rate; LAD, left atrial dimension; LVEF, left ventricular ejection fraction; RF, radiofrequecy.

Fixed C-EGMs were not detected in 39 patients with persistent AF, who received CPVI and linear ablation (group C). Patients in group C had shorter AF durations, smaller left atrial sizes and lower CHA_2_DS_2_-Vasc scores than the other two groups (Table 2). Compared with group B, with the Kaplan‐Meier analysis, the relative risk for recurrence was 0.41 in group C (95% CI 0.20–0.79, *P* = 0.009 by log‐rank test) (Fig. 7B). Besides fixed C-EGMs, hypertension and left atrial size were also associated with outcomes of ablation but the proportion of C-EGMs and non-fixed C-EGMs were not predictors of outcomes (Table 3). After adjusting for potential covariates, proportion of fixed C-EGMs independently predicted outcomes of the procedure (HR for per percent: 1.13, 95% CI 1.01–1.28, *P* = 0.038).

**Table 3:** Univariate Cox proportion-hazards regression.

Variants	HR (95% CI)	*P* value
Age (per year)	1.02 (0.98–1.08)	0.33
Sex (female as reference)	0.53 (0.24–1.18)	0.10
BMI (per kg/m^2^)	1.00 (0.91–1.11)	0.97
AF duration (per month)	1.01 (0.99–1.02)	0.11
Hypertension (yes vs. no)	2.47 (1.12–5.47)	0.03
Diabetes (yes vs. no)	1.72 (0.77–3.81)	0.19
CHD (yes vs. no)	1.62 (0.49–5.33)	0.42
Heart failure (yes vs. no)	1.37 (0.42–4.50)	0.60
eGFR (per ml/min)	0.99 (0.96–1.01)	0.24
LAD (per mm)	1.19 (1.10–1.30)	< 0.01
LVEF (per percent)	0.99 (0.94–1.04)	0.65
Proportion of C-EGMs (per percent)	1.04 (0.99–1.09)	0.08
Proportion of non-fixed C-EGMs (per percent)	1.01 (0.96–1.07)	0.65
Proportion of fixed C-EGMs (per percent)	1.22 (1.11–1.35)	< 0.01

Based on data of AF patients not receiving fixed C-EGMs ablation.

AF, atrial fibrillation; BMI, body mass index; CHD, coronary heart disease; eGFR, estimated glomerular filtration rate; LAD, left atrial dimension; LVEF, left ventricular ejection fraction.

## Discussion

The present study classified C-EGMs as fixed and non-fixed C-EGMs through local pacing. The main findings were as follows: (1) Although C-EGMs were common in patients with healthy left atrial substrate, almost all C-EGMS were not fixed during local pacing in these patients; (2) In persistent AF patients, nearly 70% C-EGMs were not fixed, but fixed C-EGMs were recorded in most persistent AF patients; (3) Compared with non-fixed C-EGMs, fixed C-EGMs had a lower amplitude, a longer duration and a longer S-P wave interval; and (4) the proportion of fixed C-EGMs rather than non-fixed C-EGMs or total C-EGMs was associated with recurrence after ablation and fixed C-EGMs ablation improved ablation outcomes.

Electrograms illustrate the electrical activity of the underlying myocardial tissue, and theoretically, C-EGMs indicate abnormal electrical activity of the underlying myocardia tissue which might precipitate arrhythmia. Recently, pieces of evidence demonstrated that AF substrates, including myocardial fibrosis, parasympathetic innervation, heterogenetic gap junction and no-pulmonary foci, were associated with c-EGMs^4–8^. Nevertheless, many non-pathological mechanisms have been recognized to make EGMs complex. In our study, the C-EGMs were commonly detected in non-AF patients, which was consistent with previous studies^9^. These results suggested that most C-EGMs might not be pathological.

Several studies demonstrated that wave collision was the major mechanism underlying C-EGMs^10,12^. The pattern of wave-front propagation determines the regions of wave-front collision. In theory, if depolarization started from the points with C-EGMs, the wave-front would propagate centrifugally and avoid wave-front collision at these points. On the contrary, if C-EGMs were caused by pathological factors, EGMs probably remain complex regardless the direction of wave propagation. However, pacing at the points of interest might depolarize the tissue beneath the recording electrodes directly which might result in artificial normalization of C-EGMs. On the other hand, it was difficult to record undisturbed EGMs at the sites of pacing. Therefore, we performed pacing adjacent to the points, which could minimize wave-front collision. Besides wave-front collision, far-field potential is another major factor making EGMs complex. Local pacing is also useful to recognize far-field potential.

In our study, in LAP patients, in whom the C-EGMs detected should be non-pathological, the morphology of almost all C-EGMs normalized after local pacing. These results supported that most non-pathological C-EGMs were not fixed during local pacing. On the other hand, in persistent AF patients, most C-EGMs were also non-fixed, which were consistent with previous studies demonstrating most C-EGMs were functional through CS pacing^12^. We found that the incidence and proportion of non-fixed C-EGMs were balanced between LAP patients and AF patients, and not associated with outcomes of AF ablation. These phenomena indicated that regions with non-fixed C-EGMs were probably not AF substrates. Therefore, it is not surprising that previous studies of C-EGMs ablation resulted in unsatisfied outcomes which might damage non-pathological atrial substrate and increase the probability of ablation related arrhythmias^16,17^.

Although most C-EGMs were not fixed, fixed C-EGMs were still detected in most AF patients but rarely in LAP patients. Compared with non-fixed C-EGMs, fixed C-EGMs had a lower voltage, a longer duration, and a longer S-P wave interval. Several AF substrates have been found to be associated with low voltage, such as atrial fibrosis, atrial wall stress and low conduction velocity^18^. Longer C-EGMs durations and S-P wave intervals analogous to S-QRS wave interval also indicated lower conduction velocity and poorly coupling to the neighboring myocardia, which might precipitate arrhythmias^10,19^. Therefore, the characteristics of fixed C-EGMs supported the concept that fixed C-EGMs rather than non-fixed EGMs might indicate AF substrate. In addition, the present study revealed that the fixed C-EGM was an independently predictor of AT/AFL/AF recurrence after ablation, which also indicates the important role of fixed C-EGMs in AF substrate.

Interestingly, in our study, the main regions encompassed by fixed C-EGMs were located on the LA anterior wall and LA septum. However, the incidence of fixed EGMs on the left atrial posterior wall was only ~ 20%, which is commonly regarded as the main source of AF. The reasons underlying the findings are unclear. However, recently, the role of the left anterior wall has garnered attention. Nedios et al. reported that in patients with AF, left atrial remodeling was more obvious in the left atrial anterior wall than posterior wall. The extent of asymmetry predicted outcomes of ablation^20^. Nakahara et al. also demonstrated that the region of the LA anterior wall compressed by the aorta was predisposed to remodeling which facilitated AF persistence^21^. Moreover, although the atrial septum is not a standard target during AF ablation, more and more evidence underlines the importance of the atrial septum in AF and atrial septum ablation acquired favorable results in some small studies^22^. In addition, we evaluated the association between C-EGMs and LVA and transitional voltage zones. The result suggested that most fixed C-EGMs overlapped with LVA and transitional voltage zones. Especially, fixed C-EGMs were more likely detected in transitional voltage zones than non-fixed C-EGMs. Yang et al. found that ablation strategy targeting C-EGMs in these regions resulted in good outcomes^14^. However, inconsistent with the previous study, there are still a significant proportion of fixed C-EGMs having normal voltage and non-fixed C-EGMs detected in LVA and transitional voltage zones which were not ablation target in our strategy.

Finally, we compared the efficacy of fixed C-EGMs ablation with linear ablation in patients with fixed C-EGMs. The results demonstrated that a significantly lower recurrence rate resulted from fixed C-EGMs ablation, even after exclusion of patients with AT/AFL recurrence. The results suggested that fixed C-EGMs eliminated the AF substrate at least in part. Furthermore, compared with linear ablation, fixed C-EGMs ablation was less time-consuming and required fewer radiofrequency applications which might potentially result in less complications of the procedure. Therefore, fixed C-EGMs ablation is expected to be an effective and safe ablation strategy in addition to CPVI.

### Limitation

The current study had several limitations. First, the ablation strategy in the participants was not randomized between linear and fixed complex EGM ablation, but was left to the operator's discretion. The outcome of ablation needed to be further confirmed by randomized controlled trials. Second, the clear mechanisms of C-EGMs were not fully elucidated in the present study. Further studies are warranted to demonstrate the pathological significance of C-EGMs, including animal studies and MRI studies, and its relation to non-pulmonary foci. Third, we only analyzed EGMs from the LA body. Although PVs and the PV antrum are the main sources of AF, we excluded these sites from analysis, because CPVI has been regarded as a standard approach to AF ablation. In addition, because of the additional time for mapping, C-EGMs mapping was not performed in the right atrium and coronary sinus, which might also harbor AF substrate^23^. Fourth, In the present study, the pacing interval was set as the RR interval during sinus rhythm minus 30 ms. Conduction velocity, a determinant of the morphology of EGMs, is usually inversely related to the heart rate. Therefore, faster pacing rate may contribute to a higher incidence of fractionated potential. Last but not least, In the present study, only one single site was pacing adjacent to the points with C-EGMs. However, the electrical conduction depends on the wavefront orientation which may influence the detection of fractionated potential. Pacing from different directions may enable to detect more C-EGMs which needs further studies using mapping tools with more electrodes assembled in different directions, such as Pentaray and HD Grid.

## Conclusion

Complex atrial electrograms recorded in sinus rhythm (C-EGMs) were common in both AF and healthy patients. C-EGMs can be classified as fixed and non-fixed during local pacing. Fixed C-EGMs might be associated with AF substrate and fixed C-EGMs ablation might improve outcomes of catheter ablation of persistent AF.

## Data Availability

The datasets used and/or analyzed during the current study available from the corresponding author on reasonable request.
